# How Robust Is Discourse Processing for Native Readers? The Role of Connectives and the Coherence Relations They Convey

**DOI:** 10.3389/fpsyg.2022.822151

**Published:** 2022-02-15

**Authors:** Mathis Wetzel, Sandrine Zufferey, Pascal Gygax

**Affiliations:** ^1^Institut de Langue et de Littérature Françaises, University of Bern, Bern, Switzerland; ^2^Department of Psychology, University of Fribourg, Fribourg, Switzerland

**Keywords:** discourse processing, connectives, online reading, polyfunctionality, frequency

## Abstract

While corpus studies have shown that discourse connectives that convey the same coherence relation can display subtle differences, research on online discourse processing has only focused on a rather limited set of connectives. Yet, different connectives – for example, rare or polyfunctional ones – might elicit different reading patterns. In order to explore this assumption, we test the robustness of discourse processing for French native speakers by measuring the way they process causal and concessive sentences that are conveyed by either an appropriate or inappropriate connective. Throughout three experiments, we change important characteristics of the connectives: we first test frequently used connectives (Experiment 1), secondly less frequent ones (Experiment 2), and finally less frequent connectives that are polyfunctional and for which different functions clearly compete (Experiment 3). Our results show that the processing for incoherent items was affected for all connectives, however readers showed altered reading fluency when infrequent connectives were used. We conclude that discourse processing is quite robust and that readers are able to insert meaning conveyed by rare connectives while still showing the highest reading ease with frequent connectives.

## Introduction

Discourse connectives are linguistic elements that give information on how to interpret the logical relations between discourse segments, such as causality or concession (Halliday and Hasan, 1976; Sanders et al., 1992). For example, by using the English connective *because*, a speaker can explicitly indicate the underlying causal relation between two clauses (example 1) by giving the instruction to interpret the second clause as the cause leading to the consequence presented in the first one.

(1) Mary cannot come to work, because she is ill.

In theoretical linguistics, this function of connectives is described as a procedural instruction and is often opposed to the conceptual meaning of words that denote a concept (e.g., Wilson and Sperber, 1998; Blakemore, 2002; Wilson, 2011). While it appears rather undisputed that the word *chair*, for example, encodes the concept of a chair and triggers a corresponding mental representation, there is still debate over how readers access and decode the procedural instructions of connectives. In Gricean models of pragmatics, the integration of procedural instructions (and correct interpretation when those are not available) is explained by inferences and contextual interpretations (Grice, 1975; see also Wilson and Sperber, 1998), whereas other pragmatic approaches explain the successful decoding of implicit relations by cognitive presuppositions (Sanders, 2005) and perceive the procedural instructions of connectives as an activator for underlying cognitive concepts (Sanders et al., 1992). In short, there are different theoretical models that discuss the procedural instructions of connectives and the way these are accessed, evaluated and handled by readers and hearers.

Using more empirical approaches, research has also investigated the extent to which connectives indicating the same coherence relation can be used interchangeably (e.g., Knott, 1996). In a recent work, Yung et al. (2021) demonstrate, for instance, that certain connectives can be (and are) used interchangeably, whereas the use of others appears to be more constrained to more specific contexts. While this study examines the subtle differences between connectives using corpus data and an offline substitution task, experimental evidence regarding the way readers *process* the procedural meaning encoded in different connectives conveying the same relation is scarcer.

So far, research on the online processing of discourse connectives has done so using connectives that are frequently used in both spoken and written language (e.g., Cain and Nash, 2011; Canestrelli et al., 2013; Asr and Demberg, 2020; Crible and Pickering, 2020; Mak et al., 2020). Yet, there are reasons to believe that different connectives might elicit different reading patterns. Research has shown, for example, that the frequency of a connective is a determining factor that explains the varying degree of understanding and mastery of connectives by teenagers and young adult speakers (Nippold et al., 1992; Zufferey and Gygax, 2020b). Aside from their frequency, the ambiguity of the connective might also affect people’s intuitions about their usage. Contrary to monofunctional connectives, ambiguous or polyfunctional connectives can convey more than one relation depending on context (e.g., *since* can convey either a temporal or causal meaning). These connectives must therefore be disambiguated before their procedural instruction can be used as a cue for discourse processing (Zamel, 1983) – a potential time consuming process that may affect the reading fluency.

In short, different connectives might affect readers’ online processing, depending on their frequency and functionality. As, to the best of our knowledge, very little research has assessed these factors for the online processing of adult readers, we attempt to fill this gap with the current study. Across three self-paced reading experiments involving the same discourse relations, we compare processing patterns of sentences linked by frequent connectives (Experiment 1), rare connectives (Experiment 2) and rare connectives that are also polyfunctional (Experiment 3). Importantly, we do not only focus on self-paced reading times for the relational segments but also measure the spill-over regions, which provides further insight into the processing patterns during integration. By doing so, we provide quantitative data that allows insight into the question of whether procedural instructions elicit the same reading patterns when they are indicated by different connectives.

## Do Rare Connectives Affect Online Processing?

Research has shown that there is high variety regarding the mastery of discourse connectives even among native speakers (e.g., Zufferey and Gygax, 2020a). One of the factors explaining this variation is the frequency of the connective. Indeed, it seems that the more often a word occurs in natural language, the better it is understood and the more it is appropriately used. For example, findings of the study by Zufferey et al. (2015) show that native speakers were able to detect in a sentence evaluation task misuses of connectives in conditional relations better than misuses of connectives in contrastive relations. The authors explained these findings by an effect of frequency, as the connective *if* in conditional relations is more frequently used than connectives such as *while* and *whereas* in contrastive relations. Furthermore, in the same task, native speakers did not score at ceiling level for the appropriate uses of the less frequent connective *while*, contrary to the appropriate uses of the more frequent connectives *if* and *when*. This led the authors to conclude that less frequent connectives can lead to less clear intuitions about their appropriate uses.

These findings resemble those by Crosson et al. (2008) who demonstrated that language minority children (i.e., children of a Spanish-speaking background living in the United States) understood cognitively simpler relations, such as additive relations, in written English better when they were conveyed by a connective that was frequently used in corpus data. The effect of frequency also applies to connectives bound to the written mode. For example, Nippold et al. (1992) showed that teenagers and young adults understood frequent connectives better than infrequent ones, this time even for more cognitively complex relations such as concessions. Similarly, Zufferey and Gygax (2020b) showed the persistent problems that young adult speakers of French have in mastering connectives that are infrequent in the spoken mode. In their experiment, participants struggled in a sentence-completion task to insert the appropriate connective, especially those that were less frequently used in corpus data. Finally, Yung et al. (2021) identified the frequency of a connective as a factor that influences the preference of a particular connective over another, especially for more simple relations in spoken language.

While all these results provide strong indications that readers benefit more from frequent connectives than infrequent ones, we hypothesize that a similar effect can be found when looking at online processing, that is, *fluency effects* while reading. As such, frequent connectives may act as a catalyst for reading fluency, whereas rare ones might be somewhat less accessible to readers.

Aside from the frequency of the connective, we believe that there is another important characteristic of connectives that can be expected to negatively affect reading fluency, namely whether connectives are more or less ambiguous in the procedural instruction they convey.

## Do Polyfunctional Connectives Affect Online Processing?

Polyfunctional connectives are connectives that can, depending on the context, convey more than one coherence relation (Crible and Cuenca, 2017). For example, the English connective *since* can either convey a causal or a temporal meaning. As seen in example (2) (taken from Zamel, 1983, p. 24), in some cases, a clear interpretation of *since* cannot be drawn without supplementary context.

(2) Since you went away, the days went cold.

Polyfunctional connectives are therefore likely to make sentences more confusing and difficult to interpret, as they have to be disambiguated before accessing the intended procedural meaning. The phenomenon of polyfunctionality is quite common, as Cartoni et al. (2013) reported that among the 100 explicit connectives annotated in the Penn Discourse Tree Bank, over two thirds were polyfunctional, in other words they had been annotated in the corpus with different sense tags.

Recently, Asr and Demberg (2020) showed, using a sentence-completion task, coherence judgment tasks and an eye-tracking experiment, the impact of polyfunctionality on reading behaviors. In their study, the authors demonstrated that although native speakers tended to use both *although* and *but* for contrastive and concessive relations, they preferred *but* to convey a contrastive relation. The study further showed that this preference could even be predicted by the distribution patterns of these connectives in discourse: by analyzing corpus data, Asr and Demberg (2020) found that *but* was indeed used more often to convey a contrastive relation.

A further indication that ambiguous connectives might affect online processing may come from research testing the processing of underspecified connectives, such as *and* when used to indicate contrastive relations (Crible and Pickering, 2020; Crible et al., 2021) or causal relations (Cain and Nash, 2011; Koornneef and Sanders, 2013), showing that inferring the intended coherence relation is not always facilitated when the connective is underspecified. However, the connectives tested in these studies (i.e., *and*, *but*, and *although*) are all rather frequently used. Consequently, there are, to our knowledge, no studies that have assessed whether polyfunctionality reinforces processing difficulty for rare connectives.

## The Present Study

In this study, we assess the influences of different French connectives on readers’ online processing. More precisely, we test whether online processing is affected when sentences contain connectives of different degrees of complexity, such as connectives that have a low frequency in the spoken mode or that are polyfunctional. In addition to that, we test the use of those connectives within two types of coherence relations (i.e., causality and concession), as we assume that connectives would have a different impact in each of them.

Causal relations can be considered cognitively simple, as they represent a form of logical continuity that readers expect from discourse (Sanders et al., 1992; Murray, 1997), and that can be even inferred without a connective (Sanders, 2005). Consequently, causal connectives play a less crucial role in maintaining discourse cohesion. Concessive relations, in contrast, are more frequently marked explicitly by a connective, as they represent a rupture of continuity (Murray, 1997). Therefore, they can be considered as more complex than causal relations and readers need to revise their expectations of continuity when encountering concessive connectives (Murray, 1997). Many studies have found that speakers not only give more correct responses in sentence completion tasks for causal relations than for concessive ones (Wetzel et al., 2020), but also process causal relations more quickly (Sanders and Noordman, 2000; Köhne and Demberg, 2013; Xu et al., 2018).

In addition to the type of coherence relation, we also focus on the way readers might identify, handle and potentially resolve incoherence due to an inappropriate use of the connectives under scrutiny. When connectives are inappropriately used, they provoke losses of coherence, which in turn should affect processing. This effect should be dependent on the type of relation as well, since – as mentioned above – concessive connectives play a more crucial role in maintaining cohesion than causal ones. In line with this idea, Murray (1997) showed that inappropriately marked concessive connectives elicited slower reading times than inappropriately marked causal or additive ones.

In sum, we assess in three self-paced reading experiments, whether potentially complicating characteristics of connectives, such as their frequency and polyfunctionality, trigger different processing patterns, or whether the readers remain unaffected by their uses and decode different coherence relations and resolve incoherence without any altered fluency. We proceed as summarized in Table 1.

**TABLE 1:** Connectives and variables tested across the three experiments.

Experiment	Marking	Relation	Type of connectives
1	Appropriate or inappropriate	Causal or concessive	Frequent
2	Appropriate or inappropriate	Causal or concessive	Rare (monofunctional)
3	Appropriate or inappropriate	Causal or concessive	Rare (polyfunctional)

### The Connectives Tested in Our Experiments

In Experiment 1, we first test two frequent connectives: the causal connective *donc* (“so”) and the concessive connective *mais* (“but”). In Experiment 2, we replace these connectives by connectives that can express the same relations but that are less frequent, namely the causal connective *ainsi* (“thus”) and the concessive connective *néanmoins* (“nevertheless”). Finally, in the third experiment, we test polyfunctional connectives that are equally rare, namely the concessive connective *or* (“however”) and the causal connective *aussi* (“therefore”).

In order to empirically assess the frequency of the connectives used in our experiments, we used the *Corpus d’étude du français contemporain* (CEFR), a resource comprising different spoken and written sub-corpora, and containing a total of 10 million words (Benzitoun et al., 2016). The normalized frequency per million words of each of the tested connectives is reported in Table 2.

**TABLE 2:** Normalized frequencies per million words of *mais*, *donc*, *néanmoins*, and *ainsi* in the written and oral corpora of CEFR.

	Spoken corpus	Written corpus	Total
*mais*	221	191	412
*donc*	221	113	334
*néanmoins*	6	20	26
*ainsi*	35	97	132
*aussi*	0 (estimated)	91 (estimated)	91
*or*	11 (estimated)	28 (estimated)	39

As can be seen in Table 2, *mais* and *donc* can be considered highly frequent connectives, whereas *néanmoins* and *ainsi* are significantly less frequent (χ^2^ = 76.93, df = 1, *p* = 2.2^*e*–16^, calculated using the *chisq.test()*-function of the stats v3.6.2 – package in *R*; R Core Team, 2020). For *or* and *aussi*, the number of occurrences had to be counted differently, as both connectives are polyfunctional, and given that the word *or* can also mean ‘‘gold’’ in French. Since we were only interested in uses of these connectives to convey cause and concession relations, we randomly selected two hundred occurrences for each connective, and counted the proportion of concessive uses of *or* and causal uses of *aussi*.^1^ We then used these proportions to estimate the total number of occurrences of these connectives in the corpus. A second Chi-square analysis revealed that both connectives have significantly fewer occurrences than *donc* and *mais* (χ^2^ = 27.21, df = 1, *p* = 1.83^*e*–07^).

There is also an important difference in terms of mono- or polyfunctionality between the connectives we evaluate. The ones used in the second experiment (i.e., *néanmoins* and *ainsi*) can be considered, following the French dictionary of connectives (Lexconn; Roze et al., 2012), as monofunctional, whereas the ones used in Experiment 3 (i.e., *aussi* and *or*) as polyfunctional, with different functions that clearly compete: *aussi* can either convey a cause-consequence relation (similar to the English “therefore”) when used in the sentence initial position or an additive relation (similar to the English “also”) when used in the sentence medial or final position. In addition to its concessive meaning, the connective *or* can also convey a background relation. This function does not seem to be lexicalized by a specific connective in English, but resembles the marker “now.”

## Experiment 1: *donc* (“So”) and *mais* (“But”)

In our first experiment, we assess the extent of which appropriate and inappropriate uses of *donc* (“so”) and *mais* (“but”) affect the online processing of causal and concessive relations. As indicated earlier, both connectives can be considered highly frequent in French. We make the following two hypotheses.

**Hypothesis 1:** Inappropriate uses of connectives should affect reading. If the signal of an inappropriately used connective clashes with the underlying coherence relation of the sentence, the resulting incoherence should lead to processing disruption (e.g., as in Lyu et al., 2020).

**Hypothesis 2:** Given that readers, by default, expect discourse to unfold in a causal manner (Sanders, 2005), they might find it easier to understand causal sentences as intended, even in the presence of a misleading (i.e., concessive) connective. Therefore, when comparing reading times for sentences containing inappropriately marked connectives, inappropriately used concessive connectives (within causal relations) might trigger less pronounced disruption effects than inappropriately used causal ones.

### Participants

We recruited 122 native French speakers (46 female) using the Internet platform *Prolific* (Oxford, United Kingdom,^2^ 2021). All participants had a minimum of 95% good ratings in previous studies on the prolific platform. Two participants were removed due to failed attention checks (i.e., high rate of inappropriate responses to verification questions, see below). The mean age of the remaining 120 participants was 29.7 years (SD = 9.3). Each participant was paid £3.15 for their participation and gave informed consent for inclusion.

### Design and Experimental Items

We used a 2 × 2 factorial design with the factors *Marking* (appropriate vs. inappropriate) and *Connective* (*mais* vs. *donc*) as within participant factors. We created 40 experimental items in French. Each item consisted of a first clause, a connective and the second clause. As seen in (3) and (4), the first clause always contained an animated subject and the second clause an anaphoric pronoun co-referring with it.

(3) *Nadia adore tous les animaux à fourrure*

“Nadia loves all furry animals”

(4) *donc elle a toujours eu un chat.*

“so she has always had a cat.”

Since having a cat can be seen as a consequence of loving furry animals, the underlying coherence relation between the two clauses is one of cause-consequence. Thus, the French connective *donc* (“so”) is an appropriate choice for this relation. In order to obtain a concessive version of the same sentence, we created another first clause, as shown in (5). This time, the connective *mais* (“but”) is the appropriate choice to convey a concessive relation. The critical segment following the connective remained constant in all conditions.

(5) *Nadia a peur des animaux à fourrure*

“Nadia is afraid of furry animals”

(6) *mais elle a toujours eu un chat.*

“but she has always had a cat.”

In order to obtain inappropriate uses of the connectives, we exchanged the connectives in the items: we used *mais* in items containing a causality and *donc* in items containing a concession. This resulted in four different versions for each item, as illustrated in Table 3 (all materials can be found at https://osf.io/96v2d/?view_only=2bd7a8bb30934f2ca02b1c7085ee5a9d).

**TABLE 3:** The four versions of an example of experimental item in Experiment 1.

Causal	Appropriate	*Nadia adore tous les animaux à fourrure donc elle a toujours eu un chat.* “Nadia loves all furry animals so she has always had a cat.”
Causal	Inappropriate	*Nadia a peur des animaux à fourrure donc elle a toujours eu un chat.* “Nadia is afraid of furry animals so she has always had a cat.”
Concessive	Appropriate	*Nadia a peur des animaux à fourrure mais elle a toujours eu un chat.* “Nadia is afraid of furry animals but she has always had a cat.”
Concessive	Inappropriate	*Nadia adore tous les animaux à fourrure mais elle a toujours eu un chat.* “Nadia loves all furry animals but she has always had a cat.”

As can be seen, the inappropriately marked concessions can, in fact, be potentially considered coherent through a process of accommodation whereas the incoherence in causes might not be easily resolved. We will come back to this difference between the two relations in section “General Discussion.”

Care was taken to ensure that the items did not contain a repetitive, parallel-structure (as in “I go on holiday because you go on holiday”) since this structure would facilitate processing (Crible and Pickering, 2020; Crible et al., 2021). In addition, for the items that contained a causal relation, further care was taken to ensure that establishing a causal link did not require a highly subjective point of view or contextual interpretation. Hence, the causal link between the first clause and the second clause was as objective and highly accessible as possible. In addition, since the French causal connective *donc* (“so”) conveys a cause-consequence relation, causality is established incrementally in a basic order. We divided each item into seven reading segments (as in Zufferey and Gygax, 2017), as seen in example (7).

(7) *Nadia adore _(1)_ / tous les animaux _(2)_ / à fourrure _(3)_ / donc-mais _(4)_ / elle a _(5)_ / toujours eu _(6)_ / un chat. _(7)_*

“Nadia loves _(1)_ / all animals _(2)_ / with fur _(3)_ / so-but _(4)_ / she has _(5)_ / always had _(6)_ / a cat. _(7)_”

After every item, participants had to answer a verification question to ensure that appropriate attention was given to the task, in the form of a statement based on the content of Segments 5–7, so that the same statement could be used in every version of the sentence. Participants had to decide whether the statement was true or false, as illustrated in (8). Half of the statements were false and half were true.

(8) *Nadia a un chien. Vrai ou faux?*

“Nadia has a dog. True or false?”

Based on results on the verification questions, two of the participants were removed from the data analysis, as they did not score at a threshold of 75% of correct responses (other participants, in mean 0.9, SD = 0.3, median = 1).^3^ In addition to our experimental items, we created 48 filler items in order to distract participants” attention from the connectives and the repetitive structure of the task. The filler items were also segmented in seven segments and contained French relative pronouns in Segment 4. The filler items were also followed by a verification question that related, contrary to the experimental items, to the first part of the sentence. This was done to ensure that participants would concentrate on all parts of the sentences, since they did not know which part of the sentence the question would address.

Four different lists of the items were created using a Latin square design to ensure that no participant would see an item twice (e.g., in the appropriate and the inappropriate version), and that all items were rotated across all conditions. Each participant was randomly assigned to one of the four lists that contained each 40 experimental items and 48 filler items. Among the experimental items, all four conditions were presented in a randomized order.

### Procedure

The experiment was conducted online *via* a weblink. A small introduction and a consent form were first presented using the online platform *Qualtrics* (Qualtrics LLC, Provo, UT, United States). The participants were then guided from *Qualtrics* to the actual experiment. The experiment was designed as a self-paced-reading-task using *PsychoPy* (Peirce et al., 2019) and hosted on the *Pavlovia* server.^4^ After reading the instructions, two training items were presented in order to familiarize the participants with the task. After completing the training, the actual experiment began. Items were presented one after the other and reading times for all segments were recorded, as well as response time to the verification question. Before every item, the participants were first asked to press the space bar in order to move on. After doing so, a red cross was presented for one second where the first segment would appear. Only one segment was presented at a time (thus allowing us to measure the reading time) and the participants moved on to the following segment by pressing the space bar. The experiment lasted approximately 30 min.

### Analysis

In order to assess whether an appropriate or inappropriate marking by frequent speech connectives conveying causal and concessive relations (*mais* “but”, *donc* “so”) influenced reading fluency, we conducted linear mixed-effects models using *R* (R Core Team, 2020) on the reading times of the sentence Segments 5–7 (the segments following the connective). We also analyzed the time needed to answer the verification questions, as this segment functioned as a wrap up region for the sentence (see Lyu et al., 2020, for similar measurements on post-critical wrap-up regions). As we were only interested in the disruptive effects of the target sentences on the time it took participants to actually reflect on them when answering the verification question, we were not really interested in dissociating right from wrong answers. As such, we analyzed all response times. Note that participants were mostly correct in their answers (for all three experiments, 90% of answers were correct on average).

We conducted linear mixed models using scaled sum contrasts for the fixed effects (Schad et al., 2020). In order to do so, we pooled all experimental conditions into one factor (i.e., containing four levels), and specified the contrasts for the main effects (and their interactions) in one contrast matrix. In order to build and apply the contrast matrix we used the *hypr* function of the hypr package (Rabe et al., 2020) and the *contrasts* function of the stats package (R Core Team, 2020). To build and run the models, we used the *lmerTest()* function of the lme4 package (Bates et al., 2015). In order to get the significance of the contributing factors of our models and interactions, we used the *summary()* function from the car package (Fox and Weisberg, 2019). We ran post-hoc tests using the *glht()* function of the multcomp() package (Hothorn et al., 2008). For all three experiments, all models contained by-item slopes of marking and by-participants slopes of connective and marking. Since we measured reading times of rather short segments, all our data contained positive skews. In order to reduce them, we first identified outliers and removed any data point above 4 s and under 50 ms, as well as any data point above 6 s and under 50 ms for the verification questions. We allowed longer times in this case because a whole sentence rather than a sentence segment was presented. As in similar literature on this subject (Crible et al., 2021), we also performed a log-transformation. These two measures reduced the skewness of our data (as tested with the *skweness()* function of the moments package, Komsta and Novomestky, 2015). After the transformation, visual evaluation indicated a normal distribution for all data^5^.

As reported in Table 4, we observe a consistent effect of marking, starting from Segment 6 onward to the response times to the verification questions.

**TABLE 4:** Experiment 1, outputs for the models of Segments 5–7 and for the response times to the verification question.

	Estimate	SE	*t*	Pr(>| *t*|)
**Segment 5**				
(Intercept)	−1.15	0.09	−128.74	<2^*e*–16^***
Connective	0.02	0.02	0.88	0.38
Marking	−0.01	0.02	−0.64	0.52
Connective: marking	−0.01	0.02	−0.58	0.56
**Segment 6**				
(Intercept)	−1.08	0.01	−119.199	<2^*e*–16^***
Connective	0.01	0.02	0.64	0.53
Marking	−0.05	0.02	−2.79	0.0057**
Connective: marking	−0.01	0.02	−0.45	0.65
**Segment 7**				
(Intercept)	−0.94	0.01	−115.57	<2^*e*–16^***
Connective	0.03	0.01	1.82	0.07.
Marking	−0.04	0.01	−2.67	0.0076**
Connective: marking	−0.03	0.01	−1.67	0.09.
**Verification question**				
(Intercept)	0.57	0.01	93.97	<2^*e*–16^***
Connective	0.016	0.01	1.28	0.20
Marking	−0.06	0.01	−4.76	2.02^*e*–06^***
Connective: marking	−0.08	0.01	−6.19	6.44^*e*–10^***

Significant codes: 0: ‘***’, 0.001: ‘**’, 0.05: ‘.’, 0.1: ‘’. Statistically significant results marked in bold.

**FIGURE 1:**
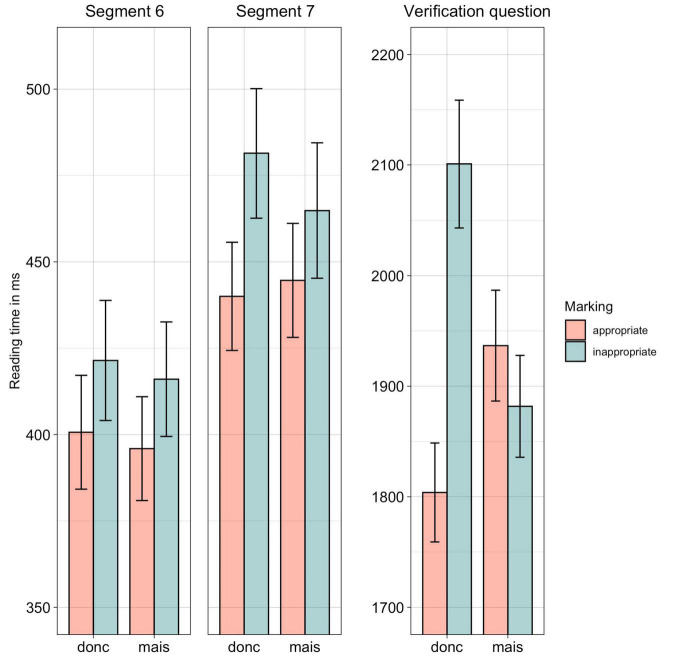
Experiment 1, reading times for Segments 6 and 7 as well as response times to the verification question. CI of 95% as error bars.

*Post-hoc* tests for the response times to the verification question further revealed that response times differed significantly when they followed sentences containing an inappropriate use of *donc* and sentences containing an appropriate use (β = 0.14, SE = 0.01, *z* = 11.17, *p* < 1^*e*–04^), whereas there was no difference between responses following sentences containing inappropriate and appropriate uses of *mais* (β = 0.02, SE = 0.01, *z* = 1.49, *p* = 0.44). Also, as can be observed in Figure 1, response times were significantly slower when the questions followed sentences containing appropriate uses of *donc* in comparison with sentences containing appropriate uses of *mais* (β = 0.07, SE = 0.01, *z* = 5.35, *p* < 1^*e*–04^).

### Discussion

In this first experiment, we examined the way readers processed sentences containing frequent connectives (*donc* “so”, *mais* “but”) that were either appropriately or inappropriately used to mark concessive and causal relations. In line with studies with a similar segment-by-segment self-paced reading procedure, we identified the final segment as being the most sensitive to potential losses of coherence (Zufferey and Gygax, 2017).

Our results show that the resulting incoherence due to inappropriate marking of connectives led to slower reading for items from Segment 6 onward to the response times to the verification questions. Participants were thus generally sensitive to losses of coherence caused by inappropriate uses of the connectives (confirming Hypothesis 1). This was especially visible for the causal connective, *donc* (“so”) most likely creating high expectations of causality that habitually facilitate reading and responses. However, we did not find a similar effect for the concessive connective for the response times to the verification questions, which suggests (following Hypothesis 2) that causality can been inferred despite the misleading concessive connective. Yet, this conclusion should be considered with caution, as incoherence might also be resolved differently in concessive relations (as already mentioned above). We will come back to this in section “General Discussion.”

Taken together, these results show that readers are highly perturbed by incoherence caused by an inappropriate connective in the wrap-up segments of sentences. Interestingly, this does seem to apply only partially to concessive connectives.

The connectives used in this experiment can be considered to be highly frequent in speech, yet, as we discussed earlier, it can be assumed that less frequent connectives may affect reading fluency (and response to verification questions) differently. Hence, in a second experiment, we assess the impact of causal and concessive connectives that are bound to the written mode (*ainsi* “thus”, *néanmoins* “nevertheless”).

## Experiment 2: *ainsi* (“Thus”) and *néanmoins* (“Nevertheless”)

In our second experiment we replicated our first experiment with connectives that are rare in the spoken mode. We make the following Hypotheses.

**Hypothesis 1:** Readers should be affected by incoherence due to inappropriately used connectives, such as in Experiment 1.

**Hypothesis 2:** However, as we are testing rare connectives this time, readers may not activate the meaning with as much ease (as they did with the connectives tested in Experiment 1). Therefore, we predict a somewhat delayed disruption effect (i.e., only apparent in the final segments of the sentence) due to inappropriately used connectives.

**Hypothesis 3:** Inappropriately used concessive connectives (within causal relations) triggered less processing disruption in Experiment 1 than causal ones. Therefore, when comparing inappropriately used (and thus misleading) connectives, causal connectives (within concessive relations) should trigger more processing disruption.

### Participants

We recruited on the Internet platform Prolific 128 native French speakers (35 female) with a mean age of 28.5 years (SD = 8.67). All participants had a minimum 95% of good ratings in previous studies. None of the participants had participated in Experiment 1 and all participants gave informed consent for inclusion. Each participant was remunerated with £3.15.

### Design and Experimental Items

We used the same materials as in Experiment 1 by replacing the connectives *mais* (“but”) and *donc* (“so”) with the connectives *néanmoins* (“nevertheless”) and *ainsi* (“thus”). These connectives convey the same relations as the connectives used in Experiment 1, yet are less frequent and bound to the written mode.

### Procedure

The procedure was the same as in Experiment 1.

### Analysis

We contrast coded linear mixed effects models using *R* using the same approach as presented in section “Analysis” in “Experiment 1: *donc* (‘So’) and *mais* (‘But’).” Once more we analyzed and transformed our data as described above.

**FIGURE 2:**
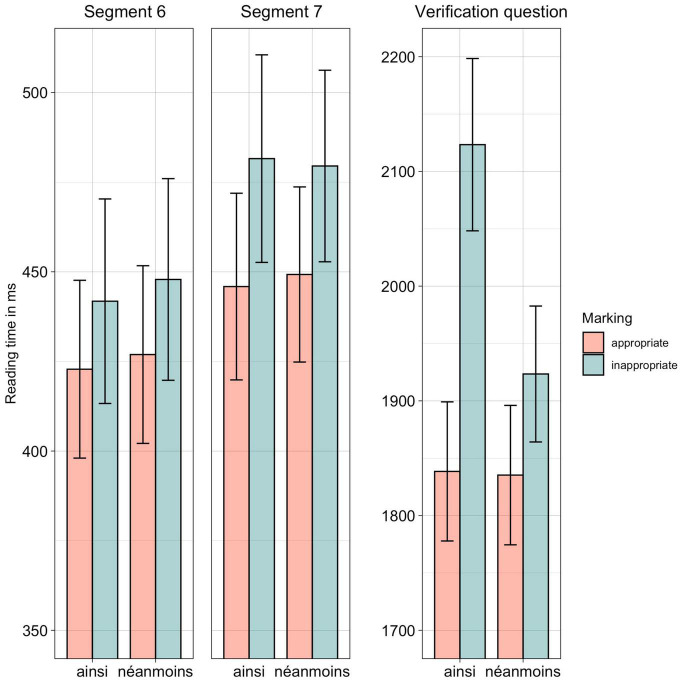
Experiment 2, reading times for Segments 6 and 7 as well as response times to the verification question. CI of 95% as error bars.

As reported in Table 5, we observed an effect of marking in Segment 7 and for the response times to the verification question. Hence, as can be also observed in Figure 2, inappropriate marking led participants to read the Segment 7 more slowly and to respond more slowly to the verification question.

**TABLE 5:** Experiment 2, outputs for the models of Segments 5–7 and for the response times to the verification question.

	Estimate	SE	*t*	Pr(>| *t*|)
**Segment 5**				
(Intercept)	−1.13	0.01	−96.92	<2^*e*–16^***
Connective	−0.01	0.02	−0.36	0.72
Marking	−0.00	0.02	−0.05	0.96
Connective: marking	0.01	0.02	0.28	0.78
**Segment 6**				
(Intercept)	−1.05	0.01	−89.94	<2^*e*–16^***
Connective	−0.01	0.02	−0.46	0.65
Marking	−0.02	0.02	−0.65	0.52
Connective: marking	−0.00	0.02	−0.13	0.90
**Segment 7**				
(Intercept)	−0.96	0.01	−89.21	<2^*e*–16^***
Connective	−0.01	0.02	−0.57	0.57
Marking	−0.06	0.02	−2.77	0.006**
Connective: marking	−0.01	0.02	−0.63	0.53
**Verification question**				
(Intercept)	0.56	0.01	70.34	<2^*e*–16^***
Connective	0.04	0.02	2.59	0.009**
Marking	−0.10	0.02	−6.25	4.70^*e*–10^***
Connective: marking	−0.50	0.02	−3.07	0.002**

Significant codes: 0: ‘***’, 0.001: ‘**’, 0.05: ‘.’, 0.1: ‘’. Statistically significant results marked in bold.

Furthermore, regarding the response times to the verification questions, *post hoc* comparisons showed a significant difference in response times between appropriate and inappropriate uses of *ainsi* (β = 0.08, SE = 0.02, *z* = 3.44, *p* < 0.005). There was however neither a significant difference between the response times to the verification questions that followed sentences containing an inappropriate or appropriate use of *néanmoins* (β = 0.03, SE = 0.02, *z* = 1.53, *p* = 0.42) nor between the appropriate uses of *néanmoins* and *ainsi* (β = 0.02, SE = 0.02, *z* = 1.06, *p* = 0.71).

### Discussion

In this second experiment, we replicated our first experiment with connectives that are less frequent than the ones tested in the first experiment, namely *ainsi* (“thus”) and *néanmoins* (“nevertheless”). The results indicate that participants were most affected by incoherence in the final sentence segment. As we observed a similar pattern in our first experiment, we conclude that less frequent connectives do not represent a general obstacle for adult readers to infer the coherence relation and that their discourse processing is quite robust, even for coherence relations that are indicated by less frequent connectives (confirming Hypothesis 1). However, reading patterns also differed slightly in comparison to the first experiment, thus showing the impact of the less frequent connectives (confirming Hypothesis 2). Contrary to Experiment 1, participants of this second experiment were only truly affected by incoherence in Segment 7 (and during the verification questions). Although incoherence equally affected reading patterns for less frequent connectives and frequent ones, readers might still find frequent connectives easier to read, as they reacted earlier to incoherence in Experiment 1.

Interestingly, and once more as observed in Experiment 1, the reaction to incoherence was especially pronounced for the causal connective, as *ainsi* led to slower response times to the verification questions when used inappropriately compared to its appropriate use (confirming Hypothesis 3). As in the first experiment we did not find this effect for the concessive connective, leading to the conclusion that the resolution of incoherence depends rather on the type of relation (i.e., cause or concession) than the type of connective that is used to convey it.

Taken together, we observed in comparison to the first experiment that the processing patterns were largely replicated, indicating that reading is rather robust, even for sentences with less frequent connectives. Yet, there were a few differences, indicating that readers may still find frequent connectives easier to process.

So far, we only assessed connectives that are monofunctional. Since little is known about whether rare connectives that are polyfunctional complicate online processing further, we extend our study with the connectives *aussi* (“therefore”) and *or* (“however”).

## Experiment 3: *aussi* (“Therefore”) and *or* (“However”)

In our third experiment, we assessed to which extent appropriate and inappropriate uses of *aussi* (“therefore”) and *or* (“however”) affect reading fluency. As discussed earlier, both connectives are rare and highly ambiguous, as both are used to indicate two very clearly different coherence relations. We make the following hypotheses.

**Hypothesis 1:** As in the two first experiments, incoherence due to inappropriately used connectives should result in processing disruption.

**Hypothesis 2:** Due to polyfunctionality of the connectives of this experiment, readers should be less sensitive toward their incorrect uses. Hence, there should be less pronounced patterns of processing disruptions for inappropriately used connectives in comparison to Experiment 2, namely no, or only smaller disruption effects in final segments.

**Hypothesis 3:** However, if effects of processing disruption for inappropriately used connectives do emerge, these effects should be especially pronounced for inappropriately used causal connectives (within concessive relations), as observed in the preceding experiments.

### Participants

We recruited 114 native French speakers (54 female) *via* the Internet platform Prolific. All participants had a minimum of 95% good ratings in previous experiments. The mean age was 28 years (SD = 8.97). None of the participants had participated in one of the two previous experiments. Each participant gave informed consent for inclusion and was remunerated with £3.15.

### Design and Experimental Items

We used the experimental and filler items from Experiment 2 and replaced the connectives with *aussi* (for the causal condition) and *or* (for the concessive condition). Note that none of the additional meanings of these connectives (additive for *aussi* and background for *or*) lead to coherent interpretations of the experiment sentences. This time, we separated both clauses with a full stop, so that both connectives were in the sentence-initial position, which represents their most common syntactic position in corpus data.

### Procedure

The procedure was the same as in Experiment 1 and Experiment 2.

### Analysis

We conducted linear mixed-effect models in which fixed effects where contrast coded, as described in section “Analysis” in “Experiment 1: *donc* (‘So’) and *mais* (‘But’).”

As reported in Table 6, we see an effect of marking in Segment 7 and for the response times to the verification question. Furthermore, we observe an interaction between the connective and the marking for the response times to the verification question.

**TABLE 6:** Experiment 3, outputs for the models of Segments 5–7 and for the response times to the verification question.

	Estimate	SE	*t*	Pr(>| *t*|)
**Segment 5**				
(Intercept)	−1.15	0.01	−139.84	<2^*e*–16^***
Connective	−0.02	0.02	−0.93	0.35
Marking	0.00	0.02	−0.2	0.84
Connective: marking	0.02	0.02	1.13	0.26
**Segment 6**				
(Intercept)	−1.08	0.01	−132	<2^*e*–16^***
Connective	.01	0.02	0.46	0.65
Marking	−0.01	0.02	−0.62	0.53
Connective: marking	0.00	0.02	0.24	0.81
**Segment 7**				
(Intercept)	−0.97	0.01	−126.35	<2^*e*–16^***
Connective	0.00	0.02	0.1	0.92
Marking	−0.04	0.02	−2.47	0.01*
Connective: marking	−0.02	0.02	−1.39	0.17
**Verification question**				
(Intercept)	0.56	0.01	93.5	<2^*e*–16^***
Connective	−0.01	0.01	−0.99	0.33
Marking	−0.02	0.01	−1.7	0.09.
Connective: marking	−0.02	0.01	−2.02	0.04*

Significant codes: 0: ‘***’, 0.01: ‘*’, 0.05: ‘.’, 0.1: ‘’. Statistically significant results marked in bold.

**FIGURE 3:**
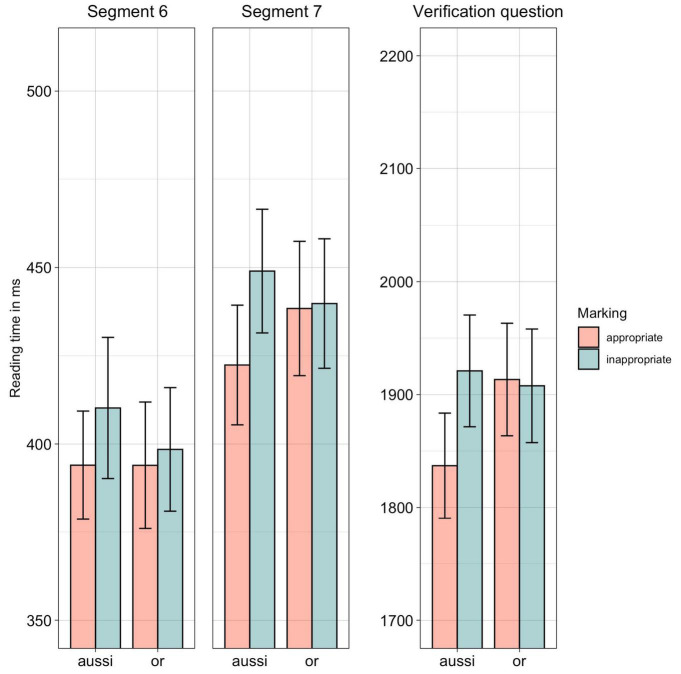
Experiment 3, reading times for Segments 6 and 7 as well as response times to the verification question. CI of 95% as error bars.

As can be seen in Figure 3, inappropriately marked sentences took longer to read than appropriate ones, yet this was independent of the relation conveyed by the sentences. *Post hoc* comparisons showed that responses to questions following appropriate uses of *aussi* differed from those following inappropriate uses of *aussi* (β = 0.057, SE = 0.012, *z* = 4.59, *p* < 0.001), and appropriate uses of *or* (β = 0.04, SE = 0.012, *z* = 3.11, *p* < 0.01). There was however no difference between the inappropriate and appropriate uses of *or* (β = −0.002, SE = 0.02, *z* = −0.16, *p* = 0.99).

### Discussion

In a third experiment, we measured reading times of causal and concessive sentences that were appropriately and inappropriately conveyed by rare and polyfunctional connectives (*aussi* “therefore”, *or* “however”). As in the previous experiments, we also measured response times to verification questions that followed the target sentences.

Results show that incoherence due to a misuse of connectives negatively affected processing times, as in the previous experiments (confirming Hypothesis 1). As in Experiment 2, less frequent (and polyfunctional), yet appropriate, connectives led to normal online processing. Yet, the reaction to incoherence was somewhat delayed, as the processing of participants was only affected by incoherence in Segment 7 and during the verification questions. As we observed a similar effect in Experiment 2, we conclude that this is caused by the lower frequency of the tested connectives. Importantly, it appears that readers were only affected by less frequent connectives generally, independently of whether the connectives were mono- or polyfunctional, as we did not observe much difference between Experiment 2 and Experiment 3 (rejecting Hypothesis 2).

In line with previous experiments, sensitivity to incoherence was especially pronounced for causal connectives, as the appropriate use of *aussi* led to faster reading and response times than its inappropriate uses. Regarding concessive connectives, we observe once more that inappropriate uses – in comparison to appropriate uses – did not lead to slower reading and response times to the verification questions. Hence, inappropriate use of *or* did not affect processing to the same extent as the inappropriate causal connective *aussi* (confirming Hypothesis 3). This finding replicates our findings from the first two experiments, indicating that this is a phenomenon related to the tested coherence relations, rather than to the individual characteristics of the tested connectives.

Finally, we also observe that appropriately marked causes led to faster response times to the verification questions than appropriately marked concessions. This confirms not only the results from Experiment 1 but also results that others have found (Sanders and Noordman, 2000; Köhne and Demberg, 2013; Xu et al., 2018), demonstrating that concession holds a higher cognitive complexity.

## General Discussion

Across three self-paced reading experiments, we assessed whether readers are affected by the frequency of connectives and the type of coherence relations they convey. In each experiment, we tested appropriate and inappropriate markings of connectives conveying causal and concessive relations. Whereas the connectives in our first experiment were common and frequently used, the connectives used in our second experiment were less frequent. In the third experiment, the connectives tested were not only less frequent, but also polyfunctional.

First of all, results show overall that reading patterns were affected by the incoherence caused by inappropriate connectives. As we observed this effect consistently across all three experiments, we conclude that readers are able, independently of the frequency or polyfunctionality of the connective, to successfully interpret the underlying coherence relation, and detect incoherencies. In the case of polyfunctional connectives this is especially remarkable, as it demonstrates the ability of readers to quickly disambiguate their meaning. Still, our results also indicate that readers show the highest appropriate vs. inappropriate processing fluency difference with frequent connectives. Regarding the detection of incoherence, findings from our first experiment demonstrate that participants were affected by incoherence already in Segment 6 – the pre-final segment containing the verb of the second clause. When tested with less frequent connectives in the second and third experiment, participants did not truly react to discrepancies until Segment 7. We conclude that while incoherence was detected in all cases, readers inferred the intended relation faster when it was signaled by a frequent connective.

The effect of incoherence was different for causal and concessive connectives. Across all experiments, the results showed that final sentence segments and verification questions primed by appropriate uses of the causal connectives were read (and answered) more quickly than those primed by its inappropriate uses. As this was independent of the characteristics of the connective (i.e., it is frequency or mono- or polyfunctionality) this finding shows that the processing of adult readers is, at least for causal connectives, robust, as readers are also able to decode causal coherence relations when they are introduced by less frequent connectives. However, response times to the verification questions following sentences with concessive connectives did not show these effects. They did not differ when they followed items either containing appropriate or inappropriate connectives. In other words, inappropriate concessive connectives did not disturb processing in a similar way to inappropriate causal ones. We would argue that readers were most likely able to infer the causal relation despite the misleading connective. Given that causal relations are cognitively simpler than concessive ones (Sanders et al., 1992) and as readers expect by default a causal continuation of a text (Sanders, 2005), the misleading concessive connective might have been more easily overridden. However, this effect could also be due to the nature of the experimental items used. For example, the incoherence of (9) can be resolved quite easily if one considers that Nadia should have, due to her love for all furry animals, more than just a cat, as illustrated in (10).

(9) Nadia loves all furry animals but she has always had a cat.

(10) Nadia loves **all** furry animals but she has always had **[only]** a cat.

In contrast, the incoherence of the item (11) cannot be easily resolved. Coherence can only be established when more context is added, as in (12).

(11) Nadia is afraid of furry animals so she has always had a cat.

(12) Nadia is afraid of furry animals so she has always had a cat **[to relieve her anxiety]**.

Hence, the contradiction between the logical link and the connective used was stronger when the inner logical link was concessive. This difference might also account for the fact that in our experiments inappropriate uses of concessive connectives did not lead to slower response times to the verification questions in comparison to their appropriate uses. Future research should more closely assess to what extent the different resolution of incoherence is an inherent feature of some coherence relations.

Finally, when comparing appropriate uses of the connectives, the results across all three experiments show a somewhat inconsistent pattern. In Experiments 1 and 3, items containing appropriately marked causes led to faster response times to the verification questions than appropriately marked concessions. This may be explained by the fact that causal relations are simpler than concessions (Sanders et al., 1992) which leads to faster processing (Sanders and Noordman, 2000; Köhne and Demberg, 2013; Xu et al., 2018). Yet, in Experiment 2 we found no difference between the response times of appropriately indicated causes and concessions. There is no obvious explanation for this difference based on our experiments. Future research will need to further assess which types of discourse connectives can reinforce or attenuate the cognitive complexity of coherence relations.

Also, further research might entail the investigation of connectives of different coherence relations. In our experiments, we tested two relations with a differing degree of continuity (i.e., concessions and causes). Yet, there may be other characteristics of connectives (and coherence relations) that might affect the way readers resolve incoherence. For instance, chronological connectives – contrary to anti-chronological ones – could also show similar effects to those observed for the continuous connectives used in our experiments. Similarly, the order of causality (basic vs. non-basic, following Sanders et al., 1992) might also affect the way readers process sentences when connectives are inappropriately used – as the insertion of non-basic causality might be more difficult when sentences are inappropriately marked.

Our results should be considered in light of some limitations. Firstly, we did not compare the reading times of sentences containing different connectives but rather focused on the way different connectives led to a different handling of incoherence (as presented and discussed in section “Experiment 1: *donc* (‘So’) and *mais* (‘But’)”). In order to directly compare different connectives, we should have tested a much larger number of experimental and filler items. Future research is therefore needed to further investigate how readers retrieve meaning from discourse despite disambiguation processes and when encountering rare linguistic elements in comparison to frequent and unambiguous ones.

Secondly, one might argue that our wrap up region – the verification questions – is too complex to truly reflect spill-over effects, all the more so, since we did not discriminate between appropriate and inappropriate responses. We would argue, however, that the observed differences in this region are meaningful as we excluded participants that scored below a threshold of 75% of correct responses and since the remaining answering rates were at ceiling level. Also, readers across all experiments were affected by incoherence when answering the verification questions, thus reflecting their difficulty in resolving the incoherence when answering the question.

Despite these potential limitations, our results show that discourse processing for adult native readers is quite robust – in terms of reading fluency –, regardless of the connective used, and that they are able to successfully infer coherence relations, be they conveyed by frequent, less frequent or polyfunctional connectives. Still, our study demonstrates that readers find it easier to access the procedural instruction of connectives that are highly frequent, as we observed delayed fluency disruptions for less frequent connectives. Regarding the theoretical descriptions of connectives, we conclude that a procedural instruction of a connective in itself can be interchanged as they similarly build coherence (thus showing the usefulness of all types of connectives), but that connectives still differ in their accessibility for readers to decode these instructions. In this regard, future research is needed to test the way readers build meaning from connectives with other characteristics (such as a differing modality) in order to deepen our understanding of the ways in which readers access and handle procedural instructions in interactions with the individual characteristics of the connective used to convey them.

## Data Availability Statement

The datasets presented in this study can be found in online repositories. The names of the repository/repositories and accession number(s) can be found below: https://osf.io/96v2d/?view_only=2bd7a8bb30934f2ca02b1c7085ee5a9d.

## Ethics Statement

The studies involving human participants were reviewed and approved by SNSF: Division I. The patients/participants provided their written informed consent to participate in this study.

## Author Contributions

MW, SZ, and PG: conceptualization. MW: data curation, investigation, methodology, resources, software, visualization, and writing—original draft. MW and PG: formal analysis. SZ: funding acquisition and project administration. SZ and PG: supervision and validation. SZ and PG: writing—review and editing. All authors contributed to the article and approved the submitted version.

## Conflict of Interest

The authors declare that the research was conducted in the absence of any commercial or financial relationships that could be construed as a potential conflict of interest.

## Publisher’s Note

All claims expressed in this article are solely those of the authors and do not necessarily represent those of their affiliated organizations, or those of the publisher, the editors and the reviewers. Any product that may be evaluated in this article, or claim that may be made by its manufacturer, is not guaranteed or endorsed by the publisher.

## Footnotes

1 We also analyzed for both connectives, aside from the first hundred occurrences with an upper case, the first hundred occurrences when written with a lower case. However, some connectives had fewer than 100 occurrences when written with an upper case (e.g., *or* with an upper case in the spoken mode only had seven occurrences), in these cases we analyzed the total number of occurrences available (hence, for *or*: 100 with lower case + 7 with upper case = 107 in total).

2
www.prolific.co

3 For the other experiments, no participants were excluded based on this threshold.

4
https://pavlovia.org

5 The data and annotated scripts for all three experiments can be found at https://osf.io/96v2d/?view_only=2bd7a8bb30934f2ca02b1c7085ee5a9d
